# Selective Interarterial Radiation Therapy (SIRT) in Colorectal Liver Metastases: How Do We Monitor Response?

**DOI:** 10.1155/2013/570808

**Published:** 2013-11-06

**Authors:** D. Hipps, F. Ausania, D. M. Manas, J. D. G. Rose, J. J. French

**Affiliations:** Hepato-Pancreato-Biliary and Transplant Surgery Unit, Freeman Hospital, Newcastle Upon Tyne NE7 7DN, UK

## Abstract

Radioembolisation is a way of providing targeted radiotherapy to colorectal liver metastases. Results are encouraging but there is still no standard method of assessing the response to treatment. This paper aims to review the current experience assessing response following radioembolisation. A literature review was undertaken detailing radioembolisation in the treatment of colorectal liver metastases comparing staging methods, criteria, and response. A search was performed of electronic databases from 1980 to November 2011. Information acquired included year published, patient numbers, resection status, chemotherapy regimen, criteria used to stage disease and assess response to radioembolisation, tumour markers, and overall/progression free survival. Nineteen studies were analysed including randomised controlled trials, clinical trials, meta-analyses, and case series. There is no validated modality as the method of choice when assessing response to radioembolisation. CT at 3 months following radioembolisation is the most frequently modality used to assess response to treatment. PET-CT is increasingly being used as it measures functional and radiological aspects. RECIST is the most frequently used criteria. *Conclusion.* A validated modality to assess response to radioembolisation is needed. We suggest PET-CT and CEA pre- and postradioembolisation at 3 months using RECIST 1.1 criteria released in 2009, which includes criteria for PET-CT, cystic changes, and necrosis.

## 1. Introduction

Selective interarterial radiation therapy (radioembolisation) is a relatively new approach to treating colorectal liver metastases in the UK. It was initially used in Australasia in 1990 [1] and has been licensed for use in Europe since 2002 [2]. Radioembolisation is used not only when conventional treatment has failed but also as first line treatment [3, 4] and it uses microspheres 20–60 microns in diameter [2]. These can be either glass or resin and are embedded with the beta emitting isotope yttrium 90. This has a half-life of 64.1 hours and decays to the stable Zirconium 90 [5].

The spheres are deployed using interventional radiology techniques via a catheter into the hepatic artery. The spheres then become lodged in the microvasculature within the tumour. Due to the small nature of the spheres, they provide interstitial high dose radiotherapy and arterial microembolisation becoming lodged in the arterioles supplying the tumour [6]. In simple transarterial embolization, the antitumour effect is via terminal artery blockade using an embolising agent such as gel foam or polyvinyl alcohol [7]. Radioembolisation as a consequence is a much less embolic procedure targeting its embolic effect further down the arterial tree and closer to the tumour leaving the surrounding liver tissue relatively intact. Experimental trials have shown that that particles of 40 microns or less have a 6–12-fold increase in chance of becoming lodged in the tumour vasculature [7] as opposed to the large particles sizes used in bland tran-sarterial embolisation.

The spheres emit high dose beta radiation to a limited area involving the tumour for an extended period of time when compared with conventional radiotherapy. The maximum penetration depth is 11 mm and the average penetration is 2.5 mm. The half-life is 64.1 hours which means that at 11 days, 94% of the isotope has decayed to infinity leaving only background radiation of no therapeutic value [2]. The consequence of these properties is that the surrounding liver tissue vascular supply and integrity remains relatively intact. The targeted approach of radioembolisation therapy is dependent on the fact that liver malignancies derive the majority of their blood supply from the hepatic artery [8]. As a result, the spheres are carried preferentially to the tumour site where they deliver the radiotherapy dose. With reference to tumour vascularity using both CT and angiography it was found that there was no statistical difference in median survival for both hypervascular and hypovascular tumours when using radioembolisation as a treatment modality, thus, both tumours are amenable to treatment [9].

The median survival for nonsurgically treated colorectal metastasis ranges from 5.7 to 19 months [10, 11]. In patients receiving no treatment, average survival is just 7.4 months [10], whilst meta-analysis of radioembolisation therapy have reported an improved survival rate of 10.8–29.4 months [4]. A randomised trial comparing radioembolisation plus chemotherapy versus chemotherapy (Fluorouracil/Leucovorin) alone showed that survival in the chemotherapy only arm was shorter (12.8 months) than the radioembolisation plus chemotherapy arm (29.4 months) [12]. A recent meta-analysis of radioembolisation as a treatment option for colorectal cancer liver metastasis showed a high “any response rate” of approximately 80% using CT response assessment for patients who had progressed on from first line conventional therapy. Furthermore, a greater than 90% response rate has been observed when radioembolisation has been used as first line therapy, as neoadjuvant to chemotherapy [4]. Potentially curative hepatic resection following downstaging/sizing by radioembolisation has been described but has only been possible in a minority of colorectal metastases cases. In the studies reviewed, hepatic resection was possible in 4 patients and has also been recorded in two separate case reports [13–15]. Radiologic complete response is rare. The highest complete response rate was 11% as judged per CT and 58% using PET. Response rate in this paper was measured using the studies' own criteria [16]. Assessing response to delivered therapies (including radioembolisation) is a crucial part of the treatment algorithm. With reference to radioembolisation, at present, a number of modalities are currently used and various criteria within these modalities are described. There appears to be no well validated standard process to recommend.

Current NICE (National Institute for Clinical Excellence) guidelines support the use of radioembolisation with systemic chemotherapy using Fluorouracil and Leucovorin for treatment of patients with hepatic metastases secondary to colorectal cancer which are not suitable for resection or ablation [17]. The Radioembolisation Brachytherapy Oncology Consortium (REBOC) recommends that candidates for radioembolisation are patients with unresectable primary to metastatic hepatic disease with liver-dominant tumour burden and a life expectancy greater than 3 months [18]. They recommend the use of radioembolisation therapy alone after failure of first line chemotherapy with Floxuridine during first line therapy or during first or second line chemotherapy as part of a clinical trial.

The aim of this paper is to review the methods used to assess response following radioembolisation in patients with colorectal liver metastases in an attempt to aid the clinician in selecting the most appropriate follow-up method.

## 2. Methods

A search of electronic databases was performed, Medline (PubMed), the Cochrane Library, Embase, and the Latin American and Caribbean Literature on Health Sciences (LILACS) from 1980 and to November 2011. Search terms based on the MeSH keywords were used, Liver Neoplasms, Colorectal Neoplasms, Neoplasm Metastasis, Yttrium Radioisotopes, Radioembolisation, Radioembolization, and SIRT.

To avoid publication bias both published and unpublished trials were identified through a computer-based search of the PubMed database. The set included randomized clinical trials, clinical trials, meta-analyses, and case series. Case reports were excluded. Papers were restricted to English language.

The search was also guided by examination of original and review article reference lists. Abstracts were not included in the analysis. Previous author's publications on the topic were excluded and only the most recent work was included.

The following information was acquired from each report: year of publication, number of patients, gender, resection status, chemotherapy regimen, modality, and criteria used to stage disease and assess response to radioembolisation, tumour markers, and overall/progression free survival.

All studies where radiological response was reported were included; although different criteria were encountered. Different study criteria for assessing tumour response available are RECIST [19], WHO [19], and EASL [20]; these are listed in Table 1. Where studies have used their own response criteria; these have been listed in Table 2.

## 3. Results

Nineteen studies evaluated the radiological/tumour marker response to radioembolisation and were used in this analysis. The studies included two randomized clinical trials, fifteen clinical trials, and three case series. Patient numbers varied from small case series up to 208 patients in the paper by Kennedy et al. [24]. All of the papers reviewed used CT as a staging method; 5 papers also used PET scan in addition to CT. Three papers were discarded as more recent studies by the same author were available [1, 12, 25]. Papers that did not distinguish outcome for colorectal metastases and other liver malignancies were excluded [26, 27]. Papers which did not list radiological response were also excluded [28].

The range of patient numbers in each study showed large variability (7–208). The total number of patients was 875. Patients evaluated in the studies had typically failed other treatment lines and were deemed to have advanced metastatic tumours not suitable for resection or ablation. Inclusion of patients with extra hepatic disease was variable; however, some studies did opt to include patients with extra hepatic disease [5, 22, 29]. Radioembolisation was also used in treatment naive patients [3]. The most common use was in conjunction with chemotherapy both systemic and local via the hepatic artery. Typical regimes used in early studies were Floxuridine, Fluorouracil, and Leucovorin. In later trials, radioembolisation was also used in the salvage setting where oxaliplatin and irinotecan had failed [15, 24].

In 10 of the 19 studies, CT was carried out at 3 months following radioembolisation. In the remaining studies it ranged from 1.5 to 6 months. RECIST (response evaluation criteria in solid tumours) criteria were used in 11/19 studies, WHO in 6/19, and local criteria in 3/19. Complete response rate ranged from 0 to 58% and a complete response rate was often not seen. Complete resolution on CT was rare; however, complete resolution was more common when assessed with PET scan 58% [16], whilst partial response varied from 0 to 90%. Stable disease was observed in 5–86% of the cases. Median survival from therapy ranged from 4.5 to 17 months.

When radioembolisation was used in treatment naive patients, 50% were partial responders compared with 28% of those patients who previously had been treated with 5FU [3]. There were however only four treatment naive patients included in the Lim et al. study [3].

A survival advantage was seen in patients who had a radiological and tumour marker response, compared to those patients that did not respond [22]. In a RCT reported by Gray et al. patients had an improved 1, 2, 3, and 5 year survival when compared with the chemotherapy only arm [30]. More recent studies used radioembolisation in the salvage setting when chemotherapy including oxaliplatin and irinotecan had failed [15, 24]. There was a significant difference in survival between responders (CR + PR + SD) and nonresponders determined by RECIST. Median survival was 16 versus 8 months, *P* = 0.0006, and survival in the responder group was 79.2% at 1 year compared with just 20.2%. At 2 years no nonresponders were alive, compared to 40.3% of the responding group. A median survival advantage was also seen in those patients who at the time of entering trials did not have extrahepatic disease (17 months versus 6.7 [31] and 37.8 months versus 13.4 [32]). Survival advantage was also seen more in patients which had previously responded to cetuximab and bevacizumab than in nonresponders [31]. Other factors noted in one paper which enrolled 133 patients to show a survival advantage were male patients (11 months versus 8 months), fewer chemotherapy cycles, and colonic site of primary versus rectal [33].

Radiological response to radioembolisation is described in Table 3.

Only 12 studies reported serum CEA levels (Table 4). These showed that there was generally a response in reduction of CEA levels (57–100%) following radioembolisation. Those who had a decrease in CEA also showed a survival advantage over those who did not, 19.1 months versus 9.3 months in one trial [31].

## 4. Discussion

The most common modality to assess response to radioembolisation was a CT scan performed at 3 months after treatment. Initial studies used the WHO staging criteria but since the publication of RECIST in 2000, more recent papers used these criteria to assess response. Response rates varied across the studies and also depended on the criteria used to judge response. Boppudi et al. showed a partial response rate of 14.8% when using the WHO definition but when the study's own criteria were applied the response rate was shown to increase to 76% [23]. This study's own response criteria to stable disease was judged as less than a 10% increase or decrease in the sum of the products of the perpendicular diameters of the index lesion. Stable disease as judged by WHO would have a less than 50% decrease or less than a 25% increase in the sum of the products of the perpendicular diameters of the index lesion [23]. This study's criteria are more lenient in judging response, this accounts for the increase in partial responders when compared with WHO criteria. Evaluation methods using necrosis and combined evaluation had overall better response rates: 45% and 50%, respectively, compared with the WHO and RECIST values of 19% and 24% [39]. Stubbs et al. reject WHO criteria for significantly underestimating response rate due to the length of time it takes for maximum size resolution to occur [22]. Instead they opt for a more lenient definition of response as listed above.

The RECIST criteria were revised in 2009 (RECIST 1.1) and this included criteria for judging PET response. It also has guidance for judging cystic changes that occur following radioembolisation. CT as judged previously may have been inaccurate due to haemorrhage, cystic degeneration, and oedema surrounding the tumour sites [16]. The other major changes to the criteria are highlighted in Table 5.

RECIST criteria are the most used of the assessment criteria, particularly given the inclusion of PET-CT scanning and guidance on the analysis of cystic changes. We would recommend the use of RECIST 1.1. In this review, all the papers using the RECIST criteria used version 1.0. In terms of timing of follow-up assessment, there is variability of between 1.5 and 6 months. Ten papers reviewed their patients at 3 months.

There has been conflicting evidence on timings of best response. Cosimelli et al. suggest that maximum response on CT scan was seen at 1.5 months [15]. Andrews et al. suggest that the parenchymal changes are most pronounced at 2 months [34], whereas Lewandowski et al. suggest best response is seen at 3-4 months in a review of interarterial treatments for hepatic malignancy [7]. This is also supported by Kennedy et al. who suggest that maximal CT and PET response occurs at 3 months [24]. Kosmider et al. showed that the best response as judged on CT varied from 0.9–50.3 months; however, the median value was 4.4 months [32] again supporting Lewandowski et al.

Although response to chemotherapy and radiotherapy is conventionally judged by change in size, tumour markers give a greater response rate [22, 24]. When CEA levels have been compared in a RCT with radioembolisation and chemotherapy versus chemotherapy alone; they have been favourable for the combined treatment of radioembolisation and chemotherapy (72% vs 47%). This is also correlated with improved survival [30].

When resection or postmortem examination has occurred, this has enabled a histological examination of the tumour; the yttrium 90 microspheres appeared as clustered eosinophil target structures [14] and the histopathological examination of the liver metastasis showed intralesional necrosis, fibrosis, and dystrophic calcifications [5, 14]. Conventional CT imaging demonstrates a difference mainly in tumour enhancement [20] which is very difficult to correlate with the histological response due to few cases where tissue is available for examination.

Few studies have used MRI to grade tumours, in the studies reviewed data was not clearly highlighted relating to MRI as an assessment tool [16, 20, 24]. It was offered as an alternative to CT as a staging tool; however, there are a number of studies that have used PET-CT scans to judge response [16, 24, 26].

PET-CT shows higher rates of partial and complete response [16, 24, 35, 39]. Unlike CT or MRI, PET-CT examines the functional element of the tumour. CEA is also a marker of metabolic function of the tumour. In one paper, all 6 PET-CT responders had a reduced level of CEA. None of these responders showed an anatomical response when measured with CT or MRI [16]. The correlation between CEA and PET-CT is also supported in another publication by Wong et al. [41]. The metabolic response is correlated with a reduction in tumour load and this is reflected by a reduced level of CEA [16]; PET-CT also proved to be more sensitive in detecting extra hepatic and hepatic lesions [16]. Where cystic changes may occur conventional cross sectional imaging such as MRI or CT may show an increase in tumour size. PET-CT however shows partial resolution and decreased activity [16, 35, 41]. Studies have shown that PET has a greater sensitivity when compared with CT in detecting recurrent disease within previously treated metastases [41]. PET-CT in one study failed to detect new lesions measuring 5–11 mm that were detected on CT scan and also in a separate study lesions detected on MRI measuring 0.5–15 mm [42]. It is reported that the sensitivity of PET is significantly lower for detection of lesions less than 1.5 cm [39]. Overall, PET shows a greater sensitivity in measuring response in direct comparison with CT; 11 lesions were deemed to have had resolution compared with just 2 on CT [16]. It correlates with the functional elements of the tumour and thus gives a better indication of tumour activity. As PET assesses the functional metastases, it can be used to assess and identify new disease, monitor response, identify residual disease, and provide the basis in terms of assessment for further salvage radiotherapy or chemotherapy as required.

With reference to PET, imaging results are based upon the measurement of SUV or standardised uptake values (SUV) which is a relative measure of [18] fluoro-D-glucose or FDG. SUV is used to account for the two most significant sources of variation that occur: patient size and the amount of FDG injected [43]. The paper by Gulec et al. recommends the use of functional tumour volume (FTV) and total lesion glycolysis (TLG) when using PET scans to assess the response to radioembolisation [44]. FTV refers to the size of the tumour that have any FDG uptake above the surrounding normal tissue uptake and the TLG was defined as the product of the functional volume and mean or maximum tumour SUV [44].

Patients found to have FTV values below 200 cc at pretreatment scans and below 30 cc at 4-week posttreatment scan were shown to have a survival advantage of greater than 12 months compared to their counterparts. Similar responses were seen in measurement of TLG in pretreatment <600 g and post treatment <100 g.

This study concluded that FTV and TLG were more informative measures of metabolic response on PET scan and results could be seen as early as 4 weeks. These results could be used as early predictors of anatomic tumour changes and a reduction in viable tumour cell volume and not just metabolic suppression. The conclusion of this paper was that FTV and TLG can be used for quantitative criteria for patient selection and disease prognostication when liver directed therapy is considered [44].

CT response was found to be highly variable which may be due to the use of concurrent and previous chemotherapy regimens used across the papers. Partial response ranged from 0% in some cases up to 90% in one paper [5]. Response was most often judged using the RECIST criteria as listed above. However, three papers used their own criteria, two rejected the WHO criteria due to underestimations of response [22, 23] and instead used more favourable study criteria to measure response.

In relation to the early response demonstrated on PET scans, changes were seen at 30 days on CT scans rather than the 3-4 month mark. These changes were in an attenuation decrease of more than 15%, and were 84.2% sensitive and 83.3% specific in predicting evaluation of response on PET scan [45]. Changes in attenuation showed higher correlation with metabolic activity of the tumour than with changes in tumour size due to composition changes such as areas of necrosis, which would show low attenuation.

The role of attenuation is in early prediction of treatment response. This would allow CT to be used earlier and more accurately in predicting response to treatment when PET scanning is not available. This information could then be used to plan a second radioembolisation procedure where further metastases exist in the opposite lobe [45]. These procedures typically take place 30–60 days afterthe initial treatment.

When PET-CT scans results and CEA levels were measured, then both showed a response, however on CT where a paradoxical increase in the size of lesions due to haemorrhage, cystic degeneration, and oedema surrounding the tumour site [16] would show progressive disease.

CEA remains a useful tool to assess metabolic function of the tumour in additions to PET-CT. PET scan can recognise the change in metabolic function. CT although the most widely used method of assessing response does not give the greatest sensitivity in measuring response when in direct comparison with PET-CT.

With regards to treatment workup and avoidance of side effects due to pulmonary or gastrointestinal complications, the paper by Denecke et al. demonstrates a suitable algorithm. They suggest first restaging the patient using CT thorax and abdomen followed by PET scan or use of PET-CT instead of individual scans. In this study, MRI followed on from PET scanning and excluded a further patients identifying lesions 0.5–15 mm. Restaging of patients using these methods streamlined patients who would benefit from radioembolisation rather than local ablative therapy.

Criteria included metastases numbers/size, which prevented local ablation, MRI showing less than 60% tumour load, no diffuse infiltration of entire organ, and liver only or liver dominant disease (extra hepatic deposits allowed if nonprogressive or no increase in size over 2–4 months) [42].

Once patients had proceeded through restaging therapy planning was commenced. This consisted of angiography and planar scintigraphy or SPECT-CT when available. The use of angiography allowed identification of target vessels and likely shunts and where necessary protective coiling is to be carried out. SPECT-CT was more accurate than CTA in predicting the distribution of microspheres and enabled further review to assess adequacy of coiling and prevent unintentional extrahepatic flow of microspheres. Following the algorithm laid out in this paper of the 13 remaining patients form the original 22 experienced no gastrointestinal or pulmonary side effects commonly seen following radiotherapy [42].

Currently, there are two large-scale trials evaluating the use of radioembolisation with chemotherapy versus chemotherapy alone as first line therapy for patients with colorectal liver metastases. The SIRFLOX trial is a multicentre trial with participating centres in Australia, The EU, New Zealand, and America. It is a prospective open labelled randomised controlled trial and compares radioembolisation with FOLFOX versus FOLFOX alone (with or without bevacizumab) [46]. The FOXFIRE trial is the UK equivalent. It is an open labelled randomised phase III trial of 5-Fluorouracil, Oxaliplatin, and Folinic acid ± interventional radioembolisation as first line treatment for patients with unresectable liver-only or liver-predominant metastatic colorectal cancer [47]. It aims to recruit 490 patients. Its primary objective will be to measure overall survival. The FOXFIRE trial will use the RECIST 1.1 staging criteria.

## 5. Conclusions

CT has previously been the assessment of choice in judging the response to radioembolisation. Although PET-CT has been shown to have limitations in detecting small metastases, this review suggests that PET-CT is a more sensitive modality. CEA remains a useful tool in assessing the functional element of a tumour and can be used to monitor response to treatment. CEA has only been measured in limited trials but it correlates with PET-CT scan results and has a role to play in determining response.

The updated RECIST 1.1 guidelines that include guidance on PET-CT should be the staging criteria of choice. The most appropriate time for assessment seems to be 3 months for both PET-CT and CT. However, using alternative measurements then the likely response can be predicted earlier with regard to using FTV and TLG for PET-CT and attenuation changes on CT scans. This early prediction has a role in further treatment planning.

In terms of use of radioembolisation for unresectable colorectal liver metastases, the greatest response was seen when used in treatment naive patients in conjunction with chemotherapy [12]. The use of radioembolisation plus HAC as a first line treatment reserves systemic chemotherapy allowing the use of systemic chemotherapy for when there is systemic disease or failure of first line treatment [22]. To avoid pulmonary and gastrointestinal morbidity, we suggest following the algorithm constructed by Denecke et al. [42].

There is no validated method to assess response that has been correlated with patient survival. However, we would recommend PET-CT pre- and postradioembolisation at 3 months, with concurrent measurements of CEA. Best response has been seen in treatment naive patients with unresectable metastases with use of HAC. The use of radioembolisation in this manner is currently supported by NICE.

## Figures and Tables

**Table 1:** Response evaluation criteria in solid tumours (RECIST) [19], world health organisation (WHO) [19], and european association for study of the Liver (EASL) [20].

	RECIST change in sum of the longest diameters	WHO change in sum of products	EASL
Complete response (CR)	Disappearance of all target lesions at 4 weeks	Disappearance of all target lesions at 4 weeks	100% necrosis of target lesions and no new lesions

Partial response (PR)	30% decrease in the Longest Diameter (LD) of target lesions at 4 weeks	50% decrease confirmed at 4 weeks	50–99% increase in necrosis

Stable disease (SD)	Neither sufficient shrinkage to qualify for PR nor sufficient increase to qualify for PD	Neither sufficient shrinkage to qualify for PR nor sufficient increase to qualify for PD	<50% increase in necrosis

Progressive disease (PD)	At least a 20% increase in the LD of target lesions; no CR, PR, or SD documented before increase	25% increase; no CR, PR, or SD documented before increase	≥25% increase in ≥1 lesion or ≥1 new lesion

CR: complete response, PR: partial response, SD: stable disease, PD: progressive disease.

**Table 2:** Individual study response criteria.

Study	Complete response %	Partial response %	Stable disease %
Anderson et al. 1992 [21]	Not measured	Not measured	Up to 25% increase or decrease in the sum of largest perpendicular diameters

Stubbs et al. 2006 [22]	Response defined as definite reduction in size of index lesions no enlarging or new lesions		No definite increase or decrease of lesion no new lesions

Boppudi et al. 2006 [23]	Not measured	Not measured	Less than a 10% change in sum of products of perpendicular diameters

**Table 3:** Radiological response.

Study	Number of patients	Extra hepatic disease allowed	Chemotherapy treatment	Radiologic staging method	Time to restaging CT (months)	Time to restaging PET (months)	Staging criteria	Complete response %	Partial response %	Stable disease %	Curative treatment %	Median survival (months)	Median survival From radioembolisation (months)
Anderson et al. [21]	7	No	Pretreatment >1 month since last course	CT	2	NA	OWN	0	0	86	NR	NR	11
Andrews et al. [34]	17	No	Pretreatment with 5FU or HAC FUDR	CT	4	NA	WHO	0	29	29	NR	NR	13.8
Gray et al. [30]	36	No	Concurrent HAC FUDR Nonprotocol also received	CT	3*	NA	WHO	6	44	28	NR	NR	17.0
Wong et al. [16]	8	Yes	Pretreatment with 5FU, LV, and Irinotecan	CT PET	3	3	WHO	CT 11 PET 58	CT 16 PET 37	CT 16 PET 5	NR	NR	NR
Lim et al. [3]	32	Yes^#^	Concurrent 5FU	CT	2	NA	RECIST	0	31	28	NR	NR	NR
Murthy et al. [29]	9	Yes	Variable pretreatment regimes	CT MR PET	NR	NR	RECIST	0	0	56	NR	24.6	4.5
Lewandowski et al. [35]	27	No	Pretreatment with capecitabine, 5FU, LV, irinotecan, or oxaliplatin	CT PET	3	3	WHO	0	CT 35 PET 83	CT 52	NR	NR	9.3
Mancini et al. [36]	35	NR	Pretreatment based on oxaliplatinor irinotecan	CT	1.5	NA	RECIST	0	12.5	75	NR	NR	NR
Kennedy et al. [24]	208	Yes^#^	Pretreatment based on oxaliplatinor irinotecan	CT PET	3	3	WHO	0	35	55	NR	NR	10.5
Stubbs et al. [22]	100	Yes	Concurrent HAC 5FU	CT	3	NA	OWN	1	73	20	NR	16.2	11
Boppudi et al. [23]	54	No^+^	Concurrent HAC 5FU	CT	3	NA	OWN WHO	9.3 1.9	76 14.8	NR	NR	NR	14.1
Jakobs et al. [37]	18	No^+^	NR	CT	2–4	NA	RECIST	0	76% any response	NR	NR	NR	NR
Sharma et al. [5]	20	Yes	Concurrent Folfox4	CT	3	NA	RECIST	0	90	10	10% (resection)	NR	9.3
van Hazel et al. [38]	23	Yes^#^	Concurrent Irinotecan	CT	3	NA	RECIST	0	48	39	13	12.2	NR
Hoffmann et al. [6]	21	No	Variable pretreatment regimes	CT	3	NA	RECIST	0	NR	NR	5% (RFA)	NR	NR
Cosimelli et al. [15]	50	Yes^★^	Pretreatment based on oxaliplatinor irinotecan	CT	1.5	NA	RECIST	2	22	24	4% (resection)	NM	12.6
Chua et al. [33]	140	Yes^◯^	NR	CT	6	NA	RECIST	1	31	31	NR	9	NR
Nace et al. [31]	51	Yes^#^	Prior treatment with various regimes	CT PET	3	3	RECIST	0	13	64			10.2
Kosmider et al. [32]	19	Yes^*⊛*^	FOLFOX or 5FU	CT	2	NA	RECIST	11	74	5	5% disease free at 6 years	29.4	10.4

NA: not applicable, NR: not recorded, HAC: hepatic artery chemotherapy, FUDR: floxuridine, 5FU: 5-fluorouracil, LV: leucovorin, FOLFOX: 5-fluorouracil, leucovorin, oxaliplatin, FOLFOX4: oxaliplatin cycle 4–12, fluorouracil, leucovorin.

*Limited by government funding, ^#^if liver dominant site of disease, ^+^if present counted as relative contraindication, ^★^limited allowed: ≤3 nodules in the same extra hepatic organ each, nodule <3 mm as assessed by a 64 slice ct [15], ^◯^limitedextrahepatic metastases at on site such as a solitary pulmonary metastases,^*⊛*^limitedpulmonary nodules or abdominal lymphadenopathy.

**Table 4:** CEA responses to radioembolisation at 3 months.

Study	Number of patients	Definition of CEA reduction	Percentage of patients (%) with CEA reduction
Gray et al. 2001 [30]	36	≥50%	72
Wong et al. 2002 [16]	8	Statistically significant	75
Murthy et al. 2005 [29]	9	NR	57
Lewandowski et al. 2005 [35]	27	50%	38
		80%	19
Kennedy et al. 2006 [24]	208	NR	70
Stubbs et al. 2006 [22]	100	82%	96
Jakobs et al. 2007 [37]	18	NR	82
Boppudi et al. 2006 [23]	54	75%	70
Sharma et al. 2007 [5]	20	NR	100*
Nace et al. 2011 [31]	51 (41 patients' levels recorded)	≥50%	41%
Kosmider et al. 2011 [32]	19	Median reduction 35%	100

NR: not recorded, *at 6 months.

The 23 patients in the van Hazel et al. [38] publication from 2009 also had a reduced CEA. Median serum CEA decreased by 82% in this trial where radioembolisation was used in conjunction with irinotecan chemotherapy.

**Table 5:** RECIST 1.0 versus 1.1 [40].

	RECIST 1.0	RECIST 1.1
Measurable disease at BL	Required, MTLS	When required then MTLS patients with nonmeasurable disease only are allowed

Minimum target lesion size	≥10 mm (Spiral CT) ≥20 mm (Conventional CT, MRI) Lymph node: not mentioned ≥20 mm (clinical)	≥10 mm (CT + MRI) ≥15 mm lymph nodes ≥20 mm chest X-ray ≥10 mm (clinical)

No. of measurable lesions	1–10 (5 per organ)	1–5 (2 per organ)

Measurement	Unidimensional	Unidimensional Lymph nodes = short axis

PD	20% increase in SLD from Nadir	20% increase in SOD + min. 5 mm increase from Nadir

Confirmation of CR and PR	After at least 28 days CR lymph node not mentioned	Only required, if response is primary endpoint and not randomized

Nonmeasurable assessment	Unequivocal progression considered as PD	(i) substantial worsening, (ii) tumour burden has increased sufficiently

Lymph node measurements	None	Specific instructions ≥15 mm, 10–14 mm, <10 mm CR lymph nodes must be <10 mm short axis

PET	Not available	May be considered to support CT, for PD and confirmation of CR

MTLS: Minimum Target Lesion Size. The size a lesion needs to be selected as a measurable target lesion at Baseline. SLD: old RECIST 1.0 sum of longest diameters for all measured target lesions' diameters to be added up at each time point. SOD: new RECIST 1.1 sum of diameters which are the longest of nonnodal lesions plus the longest of the short axis diameters of lymph nodes for measured target lesions' diameters to be added up at each time point.
